# In‐Cell NMR Spectroscopy of Functional Riboswitch Aptamers in Eukaryotic Cells

**DOI:** 10.1002/anie.202007184

**Published:** 2020-11-09

**Authors:** P. Broft, S. Dzatko, M. Krafcikova, A. Wacker, Robert Hänsel‐Hertsch, Volker Dötsch, L. Trantirek, Harald Schwalbe

**Affiliations:** ^1^ Center for Biomolecular Magnetic Resonance (BMRZ) Institute for Organic Chemistry and Chemical Biology Goethe University Max-von-Laue-Str. 7 60438 Frankfurt/M. Germany; ^2^ National Centre for Biomolecular Research Masaryk University Kamenice 5 625 00 Brno Czech Republic; ^3^ Central European Institute of Technology (CEITEC) Masaryk University Kamenice 753/5 625 00 Brno Czech Republic; ^4^ Institute of Biophysics Czech Academy of Sciences Kralovopolska 135 612 65 Brno Czech Republic; ^5^ Present address: Center for Molecular Medicine Cologne Robert-Koch-Str. 21 50931 Cologne Germany; ^6^ Center for Biomolecular Magnetic Resonance (BMRZ) Institute of Biophysical Chemistry Goethe University Max-von-Laue-Str. 9 60438 Frankfurt/M. Germany

**Keywords:** aptamers, 2′-deoxyguanosine riboswitch, HeLa cells, RNA structures, structural biology

## Abstract

We report here the in‐cell NMR‐spectroscopic observation of the binding of the cognate ligand 2′‐deoxyguanosine to the aptamer domain of the bacterial 2′‐deoxyguanosine‐sensing riboswitch in eukaryotic cells, namely Xenopus laevis oocytes and in human HeLa cells. The riboswitch is sufficiently stable in both cell types to allow for detection of binding of the ligand to the riboswitch. Most importantly, we show that the binding mode established by in vitro characterization of this prokaryotic riboswitch is maintained in eukaryotic cellular environment. Our data also bring important methodological insights: Thus far, in‐cell NMR studies on RNA in mammalian cells have been limited to investigations of short (<15 nt) RNA fragments that were extensively modified by protecting groups to limit their degradation in the intracellular space. Here, we show that the in‐cell NMR setup can be adjusted for characterization of much larger (≈70 nt) functional and chemically non‐modified RNA.

## Graphical abstract

RNA aptamers find increasing application in synthetic biology as exogenous gene control elements, but high‐resolution structural characterization in vivo is challenging. We report here the NMR‐spectroscopic observation of ligand binding by the 2′‐deoxyguanosine riboswitch aptamer in eukaryotic cells and show that the binding mode established by in vitro characterization of this aptamer is maintained in eukaryotic cellular environment.

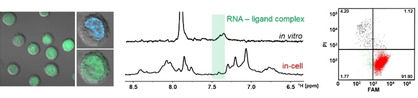

## Introduction

RNA aptamers occur in nature as ligand recognition domains of riboswitches.^[1]^ Binding of a metabolite to an aptamer domain usually modulates gene expression at the level of transcription and translation, but also other ligand binding‐induced function has been reported.^[2]^ This ability to trigger biological function by low molecular weight compounds has spurred the application of riboswitches, in particular of their aptamer domain, as artificial modules to bind small molecules with high affinity and specificity and regulate gene expression.^[3]^ Synthetic aptamers may be used for a plethora of pharmaceutical applications, such as drug delivery, biosensors^[4]^ or exogenous switches regulating transcription, translation or mRNA splicing.^[5]^ Especially bacterial riboswitches could act as precise and specific exogenous regulatory elements in eukaryotes, because they do not natively occur in them.

For rational design, high‐resolution structural data of the RNA aptamer domain and their binding capability to ligands is of great importance. X‐ray crystallography has provided numerous high resolution structures of the aptamer domain,^[6]^ in particular in their ligand‐bound state while characterization of the often induced‐fit binding mechanism has been characterized by solution NMR spectroscopy.[^7^, ^8^] Further, full‐length riboswitches and characterization of transcription intermediates has provided important insight into the kinetic mechanism of transcriptional regulation and into the potentially multistate nature of translational regulation.^[9]^

A major challenge for structural studies is the investigation of RNA aptamers under (near)‐physiological conditions. We previously demonstrated by 2D NMR that the 2′‐deoxyguanosine riboswitch aptamer is capable of binding its cognate ligand 2′‐deoxyguanosine and its non‐cognate ligand guanosine under “in‐cell‐like” in vitro conditions using bacterial cell extract as well as metabolic extract.^[10]^ The biological function of this riboswitch could be investigated in vivo using a β‐galactosidase reporter gene assay, where ligand binding was observed indirectly in bacterial cells.^[10]^

**Figure 1:**
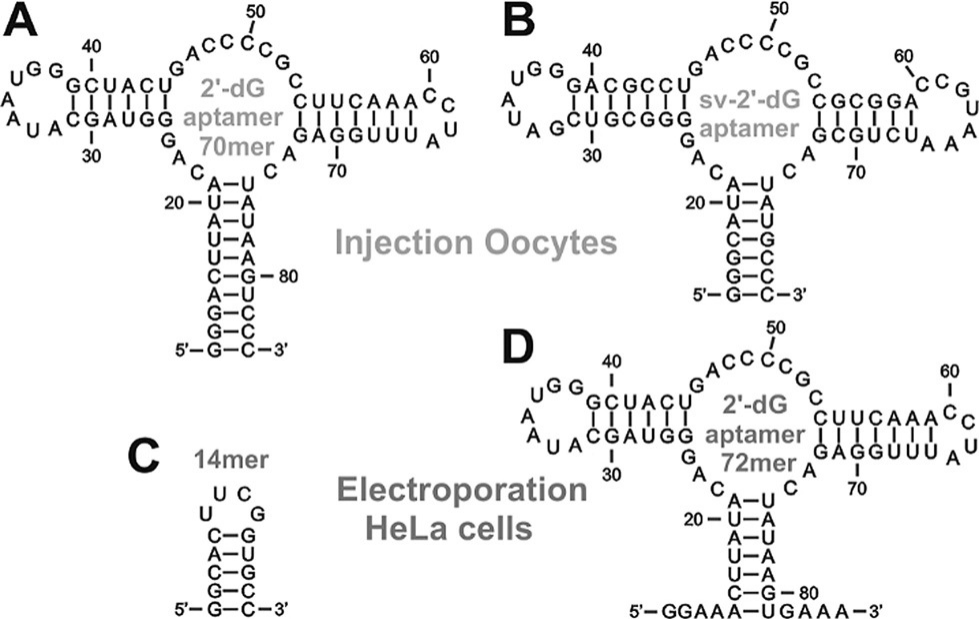
A–D) Secondary structures of 2′‐dG aptamer 70mer (A), sv‐2′‐dG aptamer (B), RNA 14mer employed as a reference for in‐cell NMR measurements (C), and 2′‐dG aptamer 72mer (D). The RNAs are delivered into the cells either by injection into oocytes (A+B) or by electroporation in HeLa cells (C+D). Ligand binding of the aptamers is mainly stabilized by Watson–Crick‐type hydrogen bonding of 2′‐dG to C74 in the three‐way‐junction of the RNA aptamer. Upon ligand binding, the closing base‐pairs A21‐U75 and G25‐U45 are stabilized and give rise to reporter imino proton signals.

Here, we seize the unique opportunity to directly test ligand binding in living eukaryotic cells, namely in *Xenopus laevis* oocytes and in HeLa cells, using state‐of‐the‐art approach of in‐cell NMR spectroscopy.[^11^, ^12^, ^13^, ^14^] A number of previous reports have focused on the NMR investigation of proteins under in‐cell conditions.[^15^, ^16^] However, reports for nucleic acids in general and of RNA in particular are rare.^[17]^ We investigate stability, structure and ligand binding capacity of an aptamer domain derived from the natural riboswitch (2′‐dG aptamer 70mer) and a Gswitch‐dGswitch chimera (sv‐2′‐dG aptamer)^[18]^ both in living oocytes and oocyte extract (Figures 1 A and B). The sv‐aptamer‐construct is a chimeric construct optimized for binding in vitro. Furthermore, we test ligand binding of a 72mer aptamer domain derived of the natural riboswitch (2′‐dG aptamer 72mer) and analyze structure and stability of a stable RNA hairpin (RNA 14mer) in living HeLa cells (Figures 1 C and D).

The sv‐2′‐dG aptamer (Figure 1 B), which is a strongly sequence‐modified RNA aptamer with high stability, can be prepared at higher concentration and contains more stable GC‐rich stems as well as more stable tertiary interactions than the 2′‐dG aptamer. To stabilize the 2′‐dG aptamer 70mer (Figure 1 A), the sequence is modified by 3 GC‐closing base pairs. The 2′‐dG aptamer 72mer (Figure 1 D) contains less GC‐rich stems than the sv‐aptamer but comes closer to the natural riboswitch in its sequence because it was only modified at the 5′‐end with an additional G, which has no stabilizing effect on the RNA structure in order to generate higher transcription yields. All three aptamers bind 2′‐deoxyguanosine in vitro and adopt a characteristic tertiary fold upon ligand binding, which is well illustrated in the imino proton region of ^1^H, ^15^N‐correlated 2D NMR spectra.^[8]^

## Results and Discussion

### In‐Cell NMR of 2′‐dG Aptamer 70mer and sv‐2′‐dG Aptamer in Oocytes

**Figure 2:**
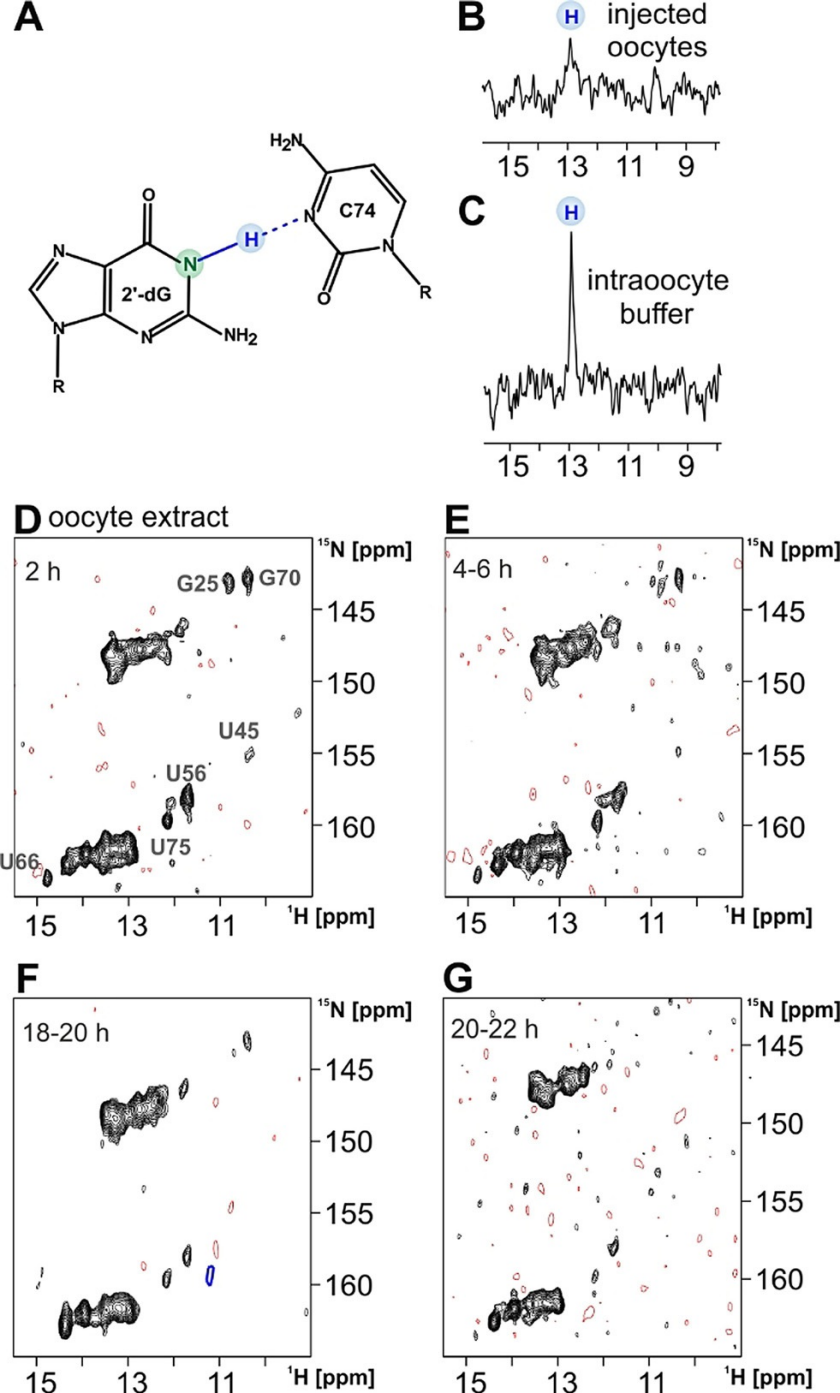
A) Watson–Crick base pair formed between C74 of the RNA aptamer domain and the ligand 2′‐deoxyguanosine. The ^15^N‐isotope of the ligand imino nitrogen is highlighted in green, the imino proton giving rise to the signal in the spectra shown in panels (B) and (C) is highlighted in blue. B+C) Comparison of ^15^N‐edited imino proton spectra of the RNA aptamer–70mer‐2′‐deoxyguanosine complex in‐cell (B) and in intraoocyte buffer (C). 60 nL of a 1.62 mm stock solution were injected into each cell, yielding ≈100 μm RNA–ligand complex per cell. The imino proton giving rise to the single signal at ≈13 ppm is marked with blue circle. The in‐cell spectrum (B) was recorded with 8192 scans and the intraoocyte buffer spectrum (C) was recorded with 2048 scans, both at *T*=291 K. For this experiment, unlabeled RNA and ^15^N‐labeled ligand has been used. D–G) Series of 2D‐spectra of the RNA aptamer–70mer‐2′‐deoxyguanosine complex in oocyte extract. The reporter signal for ligand binding, U75, is annotated in spectrum (D) and is still observed after 18 h (E). For this experiment, ^15^N‐labeled RNA and unlabeled ligand has been used.

2′‐dG aptamer 70mer in complex with ^15^N‐labeled 2′‐dG was injected into living oocytes and 1D ^15^N‐edited imino proton spectra were acquired. Ligand binding in living oocytes was observed by the characteristic H‐bond imino proton signal of 2′‐dG to C74 of the RNA (Figures 2 A, B and C). Differences in signal to noise were detected for the ^15^ N‐edited spectra (Figures 2 B and C) although both samples were measured at the same concentration. The data show that the binding mode determined in vitro is maintained in vivo. The binding mode established in vitro describes a Watson–Crick interaction between C74 and the ligand 2′‐dG, which gives rise to a characteristic imino proton signal at ≈12.8 ppm in ^1^H NMR spectra. This signal is also present in‐cell, with ^15^N editing selectively showing signals from ^15^N‐bound imino protons. Due to a high aggregation tendency of the RNA, this sample could not be prepared in concentrations sufficiently high for 2D in‐cell NMR using injection for RNA delivery. Therefore, to investigate secondary structure and stability of the 2′‐dG aptamer–ligand complex in cellular environment, we prepared oocyte cell extract as in‐cell mimic. To a good approximation, oocyte extract represents the situation inside living oocytes, at least for several hours,^[19]^ in terms of molecular composition and viscosity. Two advantages arise from the use of oocyte extract compared to intact cells: Since injection of sample into oocytes is avoided, the sample homogeneity, crucial for high quality NMR experiments, is improved, and, more importantly, the upper concentration limit of exogenously applied RNA can be markedly increased.

The tertiary fold of the RNA aptamer is conserved in the oocyte extract environment. The aptamer binds to 2′‐deoxyguanosine, forming the same tertiary structure characterized by previous in vitro structural studies.^[8]^ By recording multiple 2D spectra (Figure 2 D–G), we monitored the structural integrity of the RNA–ligand complex over time. 2 h after sample preparation, all signals are well observable. After 4–6 h, an initial loss of signal intensity is detected primarily at sites of G‐U wobble base pairs where the corresponding NMR signals are well‐resolved. G‐U base pairs are less stable than canonical base pairs. Thus, these base pairs are more susceptible to solvent exchange and correspondingly to line broadening and signal loss, especially if solvent exchange is accelerated compared to in vitro conditions. The general loss of signal intensity over time coincides with a loss of RNA structural integrity, but without significant accumulation of intermediates. This statement is supported by gel experiments showing the degradation time course of the RNA within the experimental time windows (Supporting Figure S1).

**Figure 3:**
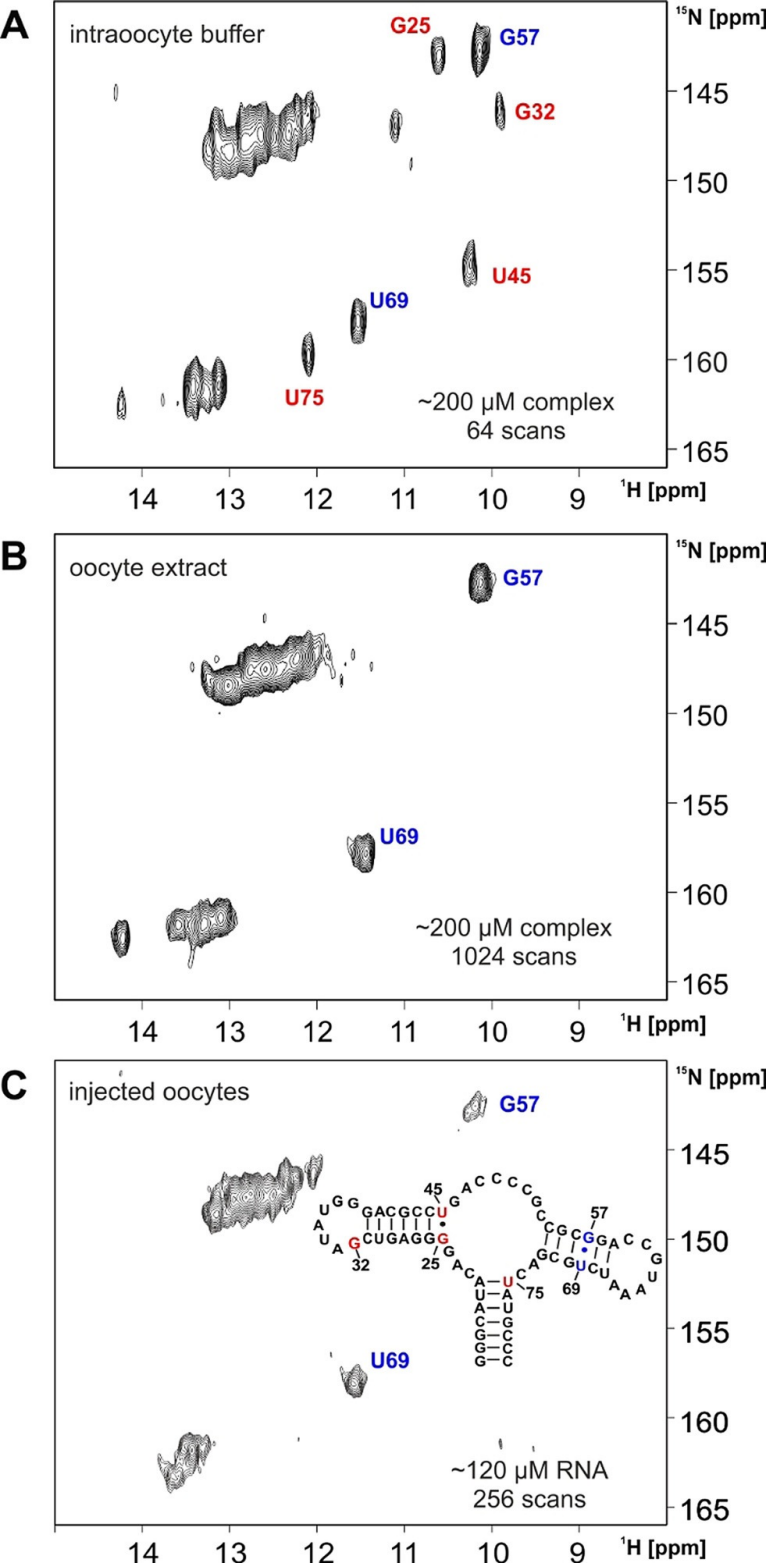
(In‐cell) ^15^N‐HMQC spectra of G, U‐^15^N‐labeled sv‐2′‐dG aptamer. The resonances of the G57 and the U69 imino protons are annotated in blue and also highlighted in blue in the secondary structure inset. The resonances of the imino protons of G25, G32, U45, and U75, which are reporter signals for ligand binding,^[8]^ are marked in red in the spectra and highlighted in red in the secondary structure inset. A) In vitro spectrum of the RNA–ligand complex (≈200 μm) in intraoocyte buffer. B) Spectrum of the RNA–ligand complex (≈200 μm) in oocyte extract. C) In‐cell spectrum of the RNA (≈120 μm) in the absence of ligand. The ^15^N‐HMQC was recorded at 700 MHz as a SOFAST‐HMQC^[20]^ employing a 2.25 ms PC9‐pulse^[21]^ centered at 12 ppm for imino proton excitation (corresponding to an excitation bandwidth of ≈1.4 kHz) and a 1.5 ms Reburp refocusing pulse^[22]^(covering ≈1.4 kHz). 1024×32 complex points were acquired in the direct and the indirect dimension with 256 scans per point and an inter‐scan delay of 0.7 s. The temperature was 291 K.

Sv‐2′‐dG aptamer could be prepared at higher concentrations than achievable for the 2′‐dG aptamer 70mer and 2D NMR spectra were recorded in living oocytes in the absence of ligand at an apparent concentration of the freely‐tumbling RNA of 100–130 μm, as estimated from the observed signal‐to‐noise in the in‐cell NMR spectra (Figure 3 C). Additionally spectra of sv‐2′‐dG aptamer in intraoocyte buffer (Figure 3 A) and in oocyte extract (Figure 3 B), both in the presence of ligand, were recorded. The aptamer in‐cell adopts the same global fold as in vitro, as evidenced by the annotated imino signal pattern in the 2D ^1^H, ^15^N correlation spectrum (Figure 3 C).

Interestingly, ligand binding for the sv‐aptamer could only be detected in vitro in intraoocyte buffer (Figure 3 A) and in vitro in potassium phosphate buffer on a G‐^15^N‐labeled RNA sample (Supporting Figure S2). G25, which is a direct reporter signal for ligand binding,^[8]^ was only visible in the in vitro spectra. The RNA–ligand complex could not be detected in oocyte extract (Figure 3 B). We suspect that the ligand‐bound form of sv‐2′‐dG aptamer could not be detected in cell extract because, on the one hand, the reporter signals are exposed to an increased exchange and, on the other hand, the K_D_ of sv‐aptamer is higher than that of aptamer 70mer, which enables near‐cognate metabolites in the cell extract to compete for the binding site, thereby preventing stable complex formation and complex detection. For the same reasons, we assume that it will not be possible to detect the complex in cells.

The lifetime of sv‐2′‐dG aptamer is sufficient for recording a 2D in‐cell spectrum. Imino proton linewidths increase considerably compared to in vitro spectra and therefore, spectral resolution is limited. We attribute the line broadening to predominantly arise from the high solvent viscosity, accompanied by molecular crowding. Still, all imino proton signals expected in the canonical region can be observed as well as the two spectrally well resolved signals of G57 and U69, arising from the G‐U wobble base pair in helix P3. In the free form of the aptamer domain, the number of detectable imino signals is limited, because a large portion of the aptamer is dynamic in the ligand‐free state. Non‐observable reporter signals include signals from U45 and U75; they become detectable only in the ligand‐bound form of the RNA aptamer, but are missing in the spectra of the free aptamer.^[8]^ It is remarkable that the free RNA aptamer domain of 67 nucleotide length maintains its secondary structure after injection into oocytes over 15 h, especially in the absence of ligand that is known to stabilize the aptamer structure.

Both the oocyte extract and the entire oocytes contain endogenous RNases and numerous other RNA‐modifying enzymes that ultimately degrade any RNA in the cell. In addition, the intracellular environment is strikingly different from in vitro conditions in terms of molecular crowding, potential unspecific interaction partners and general changes of the entire system over time. The intracellular concentration of the freely tumbling RNA aptamer of ≈100–130 μm is well above the abundance of any endogenous RNA in the cell and thus, the stability of the injected RNA may be attributed to this high concentration. In addition, the injected hybrid RNA aptamer is a sequence‐modified construct, which we originally designed for high structural stability in NMR buffer. However, even given these limitations, the stability of the intact structure of this RNA in living cells is surprising and shows that functional RNAs can in fact be characterized by 2D in‐cell NMR spectroscopy.

Encouraged by the spectral quality of 70mer RNA–ligand complex in *X. laevis* oocytes, we next aimed at obtaining spectra inside human cells. Since HeLa cells cannot be injected with RNA samples such as the large oocyte cells, the capability of electroporation for the delivery of a preformed RNA–ligand complex was investigated.

### In‐Cell NMR of RNA 14mer in HeLa Cells

Recently, Yamaoki et al. reported NMR signals of RNA in living human cells.^[12]^ They introduced solid‐phase synthesized fully 2′‐OMe modified RNA 14mer (5′‐GGCACUUCGGUGCC‐3′) into HeLa cells employing the method based on reversible membrane permeabilization using a pore‐forming toxin, Streptolysin O (SLO).^[23]^ They detected imino proton signals of RNA in the cells, but also in the outer solution of the cell suspension, which indicated that a leakage of RNA from the cells occurred during in‐cell NMR experiment. Furthermore, they showed that the in‐cell NMR sample was containing only 53 % viable RNA‐containing cells and 37 % dead cells from total cells used in the experiment. Moreover, they found that the RNA was predominantly located in the nucleus of the cells.

Here, we report our efforts to improve in‐cell NMR of RNA in human HeLa cells aiming at: a) elimination of artifacts in in‐cell NMR readout stemming from both leakage of RNA from cells in the course of in‐cell NMR spectra acquisition and high fraction of death cells in the in‐cell NMR sample (cf. Yamaoki et al.),^[12]^ and b) achieving in‐cell NMR spectra acquisition on chemically unmodified RNA. This is important since chemical modification can influence the structure of the complex RNA target. In addition, chemical modifications are difficult to introduce for larger functional RNAs.

In terms of the method improvement, we hypothesized that both, leakage of RNA from cells during NMR spectra acquisition and high cell mortality observed in original work by Yamaoki et al. (2018),^[12]^ was due to incomplete re‐sealing of toxin‐induced pores in the cell membrane. As low efficiency of toxin‐induced pores re‐sealing is inherent to this delivery method, we decided to substitute the SLO‐based delivery with an alternative approach. We accommodated the electroporation protocol originally developed by Theillet et al.^[24]^ for preparation of in‐cell NMR samples of proteins and later modified by Dzatko et al.^[13]^ and Krafcikova et al.^[14]^ for preparation of in‐cell NMR samples of DNA and DNA–ligand complexes, respectively. With the use of an adapted electroporation protocol (Material and Methods), we delivered unmodified RNA 14mer (cf. Figure 1 C) carrying a triphosphate at the 5′‐end into HeLa cells.

**Figure 4:**
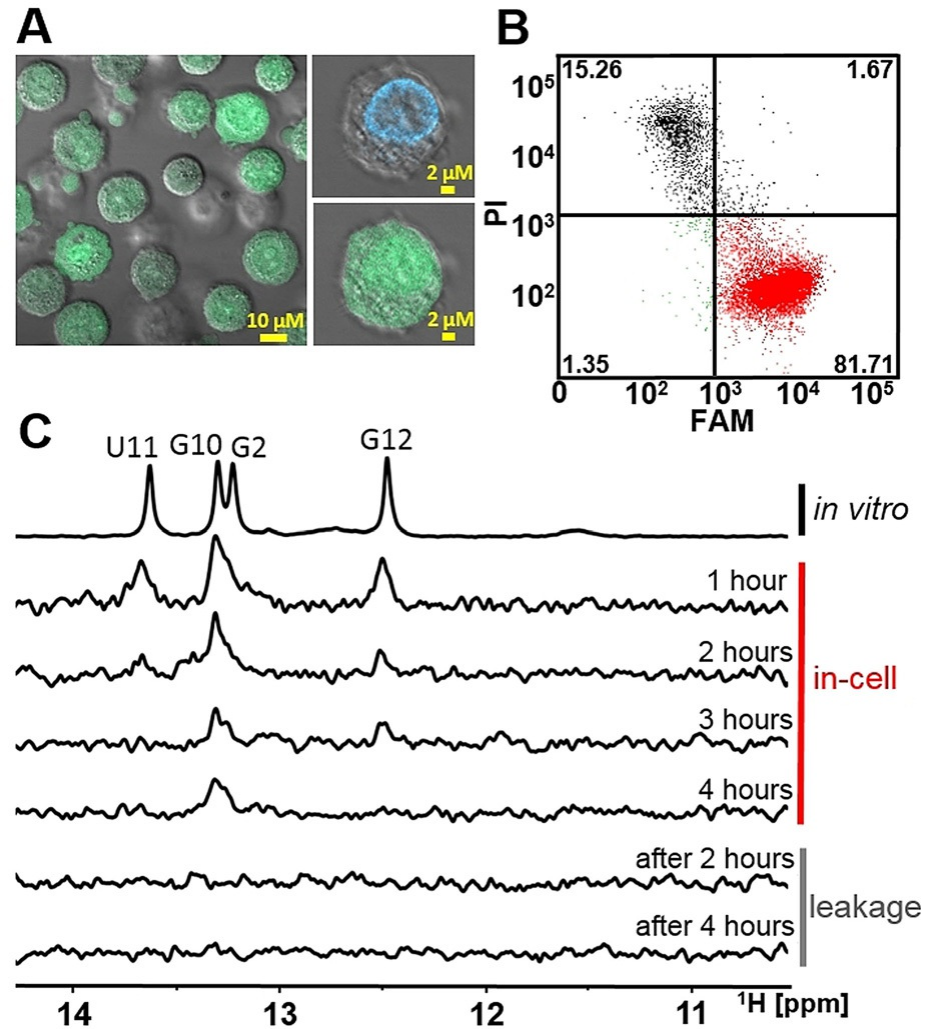
A) Confocal microscopy images of cells transfected with RNA 14mer (FAM). The green color indicates the localization of RNA 14mer (FAM). The blue color corresponds to a cell nucleus stained by Hoechst 33342. B) Double‐staining (PI/FAM) FCM analysis of transfected HeLa cells with the RNA 14mer (FAM). Percentages of a viable non‐transfected cells, viable RNA‐containing cells, non‐transfected dead/compromised cells, and transfected dead/compromised cells with RNA are indicated in left‐bottom, right‐bottom (red), left‐top, and right‐top quadrants, respectively. C) Imino region of 1D ^1^H NMR spectra of RNA 14mer in vitro (TOP) in EB‐buffer (140 mm sodium phosphate, 5 mm KCl, 10 mm MgCl_2_, pH 7.2) and corresponding spectrum of HeLa cells transfected with RNA 14mer (FAM) (MIDDLE). Imino region of 1D ^1^H NMR spectrum of extracellular fluid (supernatant) taken from the in‐cell NMR samples after completion of the spectra acquisition (BOTTOM). The (in‐cell) NMR spectra were acquired at 20 °C.

To allow monitoring the transfection efficiency and localization of transfected RNA in the cells with flow cytometry (FCM) and confocal microscopy, the unlabeled RNA was supplemented with 5′‐FAM‐labeled RNA (in a ratio of 40:1). The confocal microscope images of transfected cells indicated that the delivered RNA was localized in both, cell nucleus and cytosol, and was homogeneously dispersed all over the cell (Figure 4 A). Yet, FCM analysis performed after the in‐cell NMR spectra acquisition (total time of 4 hours), showed that the RNA transfection efficiency and viability of the transfected cells were higher compared to those achieved by SLO‐based method: More than 80 % of RNA transfected cells were viable (Figure 4 B) compared to 53 % of viable and transfected cells observed by Yamaoki et al.^[12]^ after 1 hour in‐cell NMR measurement. Notably, out of cells contributing to in‐cell NMR signal (cells in right‐bottom and right‐top quadrants of FCM plot, Figure 4 B) 98 % were viable. Noteworthy, we were able to deliver the RNA 14mer into HEK and RPE cells with similar results (Supporting Figure S3).

1D ^1^H in‐cell NMR spectra of electroporated HeLa cells were recorded in a time window of 4 hours to monitor RNA stability in the intracellular space (Figure 4 C). The in‐cell NMR experiment showed characteristic imino proton signals of RNA 14mer after 1 hour measurement time when comparing with the in vitro spectrum of the RNA in EB‐buffer. The quality of the in‐cell NMR spectrum in terms of signal‐to‐noise ratio and resolution was comparable to corresponding in‐cell NMR spectrum of fully 2′‐OMe modified RNA 14mer reported by Yamaoki et al.^[12]^ Both SLO‐ and electroporation delivery methods appear to provide an intracellular concentration of delivered RNA in the same range, i.e., between 5–15 μm.

The signal intensity after 1 hour measuring was set to 100 %. With increasing measurement time, unspecific degradation of the RNA was detected. After 4 hours measurement time the signal intensity dropped to 26 %. The data showed that due to degradation of the RNA in the cells the time window for measuring in‐cell NMR of RNA should be as short as possible and not extend 2 hours, where 77 % of the imino proton signal intensity was still detectable. To check whether RNA leakage from cells took place, the supernatant of the cell suspension was measured after 2 and 4 hours of the in‐cell NMR sample. There was no leakage of RNA from the cells detected.

The data thus suggest that compared to SLO‐based approach the electroporation‐based delivery of RNA not only improves parameters of in‐cell NMR sample in terms of both absence of RNA leakage in the course of in‐cell NMR spectra acquisition and increased viability of the cells, but that it can be applied to distinct cell lines.

### In‐Cell NMR of 2′‐dG Aptamer 72mer in HeLa Cells

**Figure 5:**
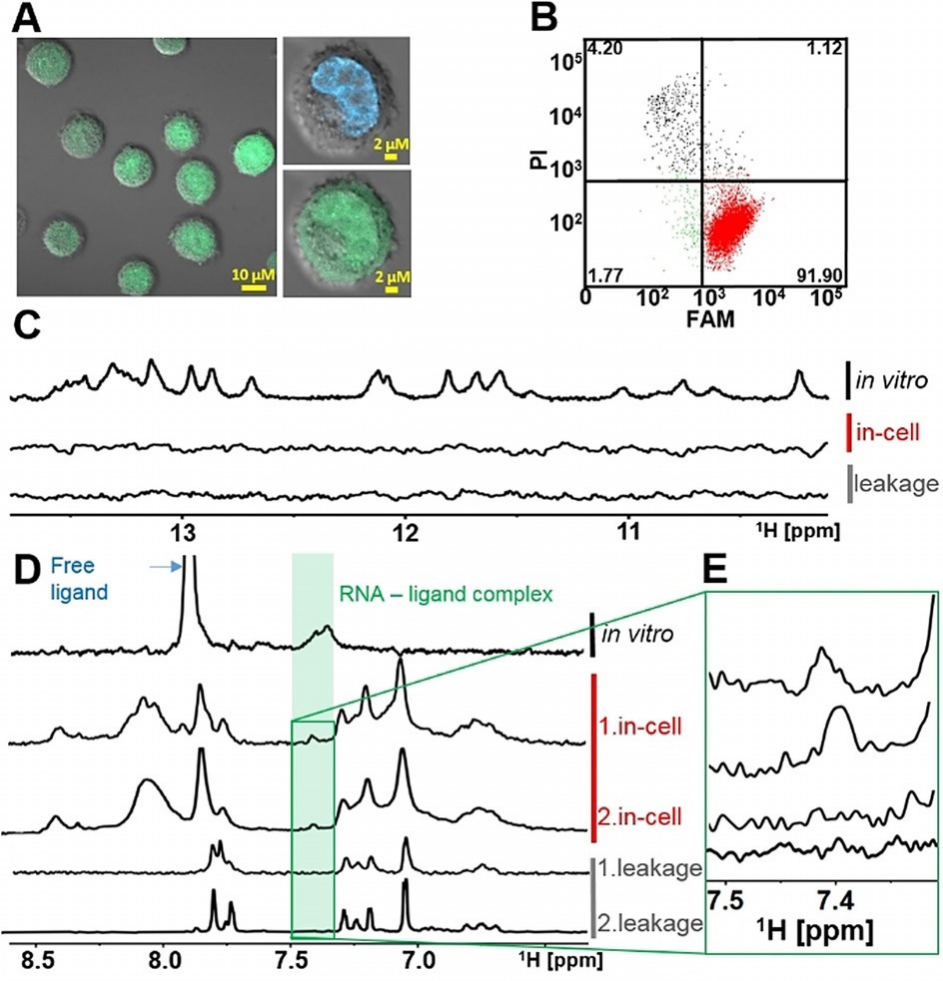
A) Confocal microscopy images of cells transfected with aptamer–ligand complex. The green color indicates the localization of (FAM)‐aptamer/(FAM)‐aptamer–ligand complex. The blue color corresponds to a cell nucleus stained by Hoechst 33342. B) Double‐staining (PI/FAM) FCM analysis of transfected HeLa cells with the aptamer–ligand complex. Percentages of a viable non‐transfected cells, viable aptamer–ligand complex containing cells, non‐transfected dead/compromised cells, and transfected dead/compromised cells with aptamer–ligand complex are indicated in left‐bottom, right‐bottom (red), left‐top, and right‐top quadrants, respectively. C) Imino region of 1D ^1^H NMR spectra of aptamer–ligand complex in vitro (TOP) in EB‐buffer (140 mm sodium phosphate, 5 mm KCl, 10 mm MgCl_2_, pH 7.2) and corresponding spectrum of HeLa cells transfected with aptamer–ligand complex (MIDDLE). Imino region of 1D ^1^H NMR spectrum of extracellular fluid (supernatant) taken from the in‐cell NMR samples after completion of the spectra acquisition (BOTTOM). D) 1D ^13^C‐edited NMR spectra of the aptamer–ligand complex in vitro in EB‐buffer (TOP) and corresponding spectra of HeLa cells transfected with aptamer–ligand complex. Note: The NMR spectra from two independent experiments are provided (MIDDLE). 1D ^13^C‐edited NMR spectra of extracellular fluid (supernatant) taken from the respective in‐cell NMR samples after completion of the spectra acquisition (BOTTOM). E) Zoomed region of 1D ^13^C‐edited NMR spectra from D). Note: For confocal microscopy images and FCM analysis from the second in‐cell NMR experiment, see Supporting Figure S4. The (in‐cell) NMR spectra were acquired at 20 °C.

**Figure 6:**
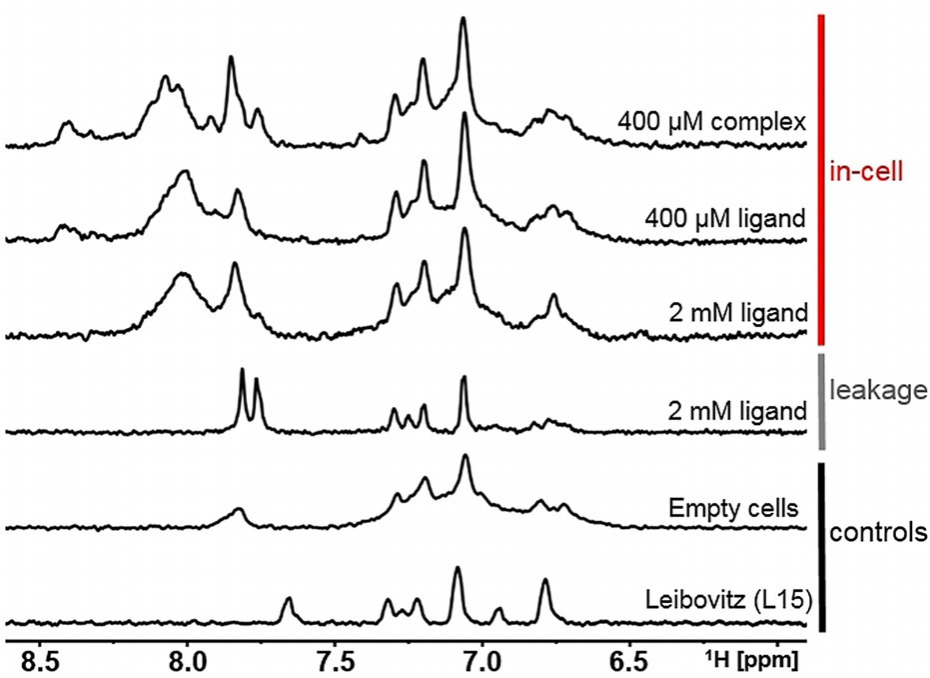
1D ^13^C‐edited in‐cell NMR spectra of cells transfected with aptamer–ligand complex (400 μm aptamer/2 mm 2′‐deoxyguanosine), cells transfected with 400 μm and 2 mm ligand, and non‐transfected cells. 1D ^13^C‐edited in vitro NMR spectra of extracellular fluid taken from the 2 mm 2′‐deoxyguanosine in‐cell NMR sample after completion of the spectra acquisition (leakage), and Leibovitz L15 medium. The (in‐cell) NMR spectra were acquired at 20 °C.

Using the electroporation procedure described above, we introduced the preformed aptamer–ligand complex, consisting of 2′‐dG aptamer 72mer and its ^13^C, ^15^N labeled ligand 2′‐deoxyguanosine into HeLa cells. FCM analysis showed that after electroporation more than 90 % of the cells were viable RNA containing cells and less than 6 % of the cells were either dead or had compromised cell membrane integrity (Figure 5 B). Confocal microscopy indicated that the RNA was homogeneously dispersed all over the cell (Figure 5 A). While inherently broader (compared to RNA 14mer) imino signals from 72nt aptamer/aptamer–ligand complex were below detection limit (Figure 5 C), the signals of unbound (free) and aptamer bound ^13^C‐labeled ligand were observed in the ^13^C‐edited in‐cell NMR spectrum (Figure 5 D). The signal at 7.4 ppm (in Figure 5 D highlighted in green) showed that the aptamer–ligand complex was present in the cells. After completion of data acquisition the extracellular fluid of the in‐cell NMR sample was measured. Neither the in‐cell proton spectra nor the in‐cell ^13^C‐edited spectra showed any leakage of aptamer/aptamer–ligand complex from the cells. A leakage of unbound ligand was detected in the ^13^C‐edited spectra. To ensure that the above mentioned signal at 7.4 ppm stems from bound ligand in the complex, we carried out the in‐cell experiment twice (for confocal microscope images and FCM analysis from the second in‐cell NMR experiment see Supporting Figure S4). The signal can reproducibly be detected as strong signal (Figure 5 E). To determine the origin of the additional signals in the ^13^C‐edited in‐cell NMR spectrum of aptamer–ligand complex, in cell spectra of cells electroporated with 400 μm ligand and 2 mm ligand, and a spectrum of non‐transfected cells and extracellular fluid (Leibovitz L15 medium) were measured (Figure 6).

The ^13^C‐edited in‐cell NMR spectra of the ligand showed that the ligand was bound to cellular off‐targets in the cell and caused additional signals above 8 ppm and was also contributing to the overall spectrum in the region between 7.7–7.9 ppm. Moreover, the spectrum of non‐transfected cells and Leibovitz L15 medium showed that additional signals between 6.6–7.4 ppm and 7.6–7.9 ppm in the ^13^C‐edited complex spectrum were originating from background (natural abundance) of both, cells and Leibovitz L15 medium. The signals in the range from 7.7–8.0 ppm of the in‐cell spectrum of the complex (and leakage) (Figure 5 D) were the result of “off‐target” interactions of the ligand with cellular (medium) components (cf. in vitro spectrum of free ligand Figure 5 D (top) vs. in‐cell and leakage spectra of the ligand Figure 6). In this regard, the difference observed in the region between 7.7–8‐0 ppm between the in‐cell spectrum of the complex and the in‐cell spectrum of 2′‐deoxyguanosine alone can be attributed to altered binding of the ligand to cellular (medium) “off‐targets” in the presence of the RNA, which acts as a competitor of these “off‐target” interactions.

However, the differences in the region highlighted in green (Figure 5 D) of the spectra have different origin. The signal in in vitro spectrum of the complex corresponding to the bound form of the ligand is broad and display two apparent maxima, while the respective signal in the in‐cell spectrum of the complex is narrow and displays one maximum only. We attribute this difference to increased structural heterogeneity of the binding site under in vitro conditions compared to the situation in cells.

After completion of spectra acquisition the supernatant of the 2 mm ligand in‐cell NMR sample was measured. The spectrum showed leakage of the ligand. However, FCM analysis showed that more than 90 % of the cells were viable after transfection with 400 μm/2 mm ligand (Supporting Figures S5A and S5B) which was also supported by confocal microscope images taken after transfection with ligand, which showed intact cells (Supporting Figure S4C), indicating that the ligand was not toxic for the cells.

## Conclusion

In this report, we extend in‐cell NMR spectroscopy for RNA both in terms of methodology as well as complexity of the detected RNA system. By detecting a functional riboswitch aptamer domain bound to its native ligand in living oocytes and in viable human HeLa cells, we demonstrated the applicability of in‐cell NMR on non‐modified RNA, which proved to maintain structural integrity long enough to allow for recording of NMR spectra. It should be particularly emphasized that the binding mode of this prokaryotic riboswitch aptamer determined by means of in vitro studies was identical in eukaryotic cells, although prokaryotic and eukaryotic cells as well as in vitro conditions differ with regard to their composition. In contrast to in‐cell studies in bacteria, which showed that the cellular milieu can have influence on the protein conformation and stability,^[16]^ our studies showed that this is not the case for the 2′‐deoxyguanosine‐sensing riboswitch in eukaryotic cells.

The interference from cellular background represents one of the major problems in in‐cell NMR studies of biomolecules including RNA. In principle, the site‐specific labeling of the RNA with 100 %‐abundant ^19^F nuclei might provide solution to this issue. With ^19^F labeling of the RNA one can obtain background free in‐cell spectra because biological molecules do not contain fluorine atoms. A number of ^19^F RNA labeling strategies were developed for monitoring of RNA structural transitions and interactions in vitro in past two decades.^[25]^ However, with a single exception, none of these strategies was tested in cells thus far. In 2017, Bao et al.^[26]^ showed that a covalent tagging of 5′‐terminus of telomeric G‐quadruplex forming RNA with six‐ethyl linked 3,5 bis(trifluoromethyl)phenyl moiety allowed ^19^F in‐cell NMR readout on the RNA G‐quadruplex structure. Whether this strategy is applicable to in‐cell NMR studies of other (non‐G‐quadruplex) RNAs is not currently known.

For oocyte cells, in‐cell RNA concentrations of ≈120 μm can be reached via micro‐injection, which offers sensitivity almost comparable to in vitro conditions. Furthermore, oocytes remain viable at 18 °C for several hours, thus enabling long (2D) NMR experiments. However, inside the HeLa cells more relevant to the human physiology, the intracellular concentration that can be reached by available transfection methods is around 10 μm. Due to combined effects of RNA degradation, low intracellular RNA concentration, and fast relaxation of NMR signals inherently encountered for large RNAs (>50 nt) the acquisition of multi‐dimensional in‐cell NMR spectra is impractical, but the parameters of in‐cell NMR sample are sufficient for observing RNA fingerprint resonances on isotope‐labeled sample.

Detecting ligand binding to an exogenously prepared prokaryotic riboswitch in two different eukaryotic cells provides substantial and direct support for Systems Biology application where bacterial riboswitch typically not existing in eukaryotes can fundamentally be utilized as exogenous regulation element. Further, riboswitch RNA with submicromolar affinities to their cognate ligand can also be utilized as structural readout for ligands and changes in their concentration in eukaryotic cells. Careful tailoring of aptamer and cellular system, in‐cell studies of RNA are thus feasible utilizing methodology outline here.

## Conflict of interest

The authors declare no conflict of interest.

## Supporting information

As a service to our authors and readers, this journal provides supporting information supplied by the authors. Such materials are peer reviewed and may be re‐organized for online delivery, but are not copy‐edited or typeset. Technical support issues arising from supporting information (other than missing files) should be addressed to the authors.

Supplementary

## References

[1] M. Mandal , B. Boese , J. E. Barrick , W. C. Winkler , R. R. Breaker , Cell 2003, 113, 577. 12787499 10.1016/s0092-8674(03)00391-x

[2] L. Bastet , P. Turcotte , J. T. Wade , D. A. Lafontaine , RNA Biol. 2018, 15, 679. 29537923 10.1080/15476286.2018.1451721 PMC6152446

[3] A. D. Ellington , J. W. Szostak , Nature 1990, 346, 818. 1697402 10.1038/346818a0

[4] S. Findeiß , M. Etzel , S. Will , M. Mörl , P. F. Stadler , Sensors 2017, 17, 1990. 10.3390/s17091990 PMC5621056 28867802

[5a] A. Wittmann , B. Suess , FEBS Lett. 2012, 586, 2076; 22710175 10.1016/j.febslet.2012.02.038

[5b] P. Machtel , K. Bąkowska-Żywicka , M. Żywicki , J. Appl. Genet. 2016, 57, 531. 27020791 10.1007/s13353-016-0341-x PMC5061826

[6a] A. Serganov , Y.-R. Yuan , O. Pikovskaya , A. Polonskaia , L. Malinina , A. T. Phan , C. Hobartner , R. Micura , R. R. Breaker , D. J. Patel , Chem. Biol. 2004, 11, 1729; 15610857 10.1016/j.chembiol.2004.11.018 PMC4692365

[6b] A. D. Garst , A. Héroux , R. P. Rambo , R. T. Batey , J. Biol. Chem. 2008, 283, 22347; 18593706 10.1074/jbc.C800120200 PMC2504901

[6c] J. Buck , J. Noeske , J. Wöhnert , H. Schwalbe , Nucleic Acids Res. 2010, 38, 4143; 20200045 10.1093/nar/gkq138 PMC2896527

[6d] C. W. Reiss , S. A. Strobel , RNA 2017, 23, 1338. 28600356 10.1261/rna.061804.117 PMC5558903

[7a] J. Noeske , J. Buck , B. Fürtig , H. R. Nasiri , H. Schwalbe , J. Wöhnert , Nucleic Acids Res. 2007, 35, 572; 17175531 10.1093/nar/gkl1094 PMC1802621

[7b] M. Kang , R. Peterson , J. Feigon , Mol- Cell 2009, 33, 784; 19285444 10.1016/j.molcel.2009.02.019

[7c] A. K. Weickhmann , H. Keller , E. Duchardt-Ferner , E. Strebitzer , M. A. Juen , J. Kremser , J. P. Wurm , C. Kreutz , J. Wöhnert , Biomol. NMR Assignments 2018, 12, 329. 10.1007/s12104-018-9834-3 30051308

[8] A. Wacker , J. Buck , D. Mathieu , C. Richter , J. Wöhnert , H. Schwalbe , Nucleic Acids Res. 2011, 39, 6802. 21576236 10.1093/nar/gkr238 PMC3159443

[9a] J. K. Wickiser , W. C. Winkler , R. R. Breaker , D. M. Crothers , Mol. Cell 2005, 18, 49; 15808508 10.1016/j.molcel.2005.02.032

[9b] A. Haller , M. F. Soulière , R. Micura , Acc. Chem. Res. 2011, 44, 1339; 21678902 10.1021/ar200035g

[9c] H. Steinert , F. Sochor , A. Wacker , J. Buck , C. Helmling , F. Hiller , S. Keyhani , J. Noeske , S. Grimm , M. M. Rudolph et al., eLife 2017, 6, 0e21297; 10.7554/eLife.21297 PMC5459577 28541183

[9d] C. Helmling , D.-P. Klötzner , F. Sochor , R. A. Mooney , A. Wacker , R. Landick , B. Fürtig , A. Heckel , H. Schwalbe , Nat. Commun. 2018, 9, 944. 29507289 10.1038/s41467-018-03375-w PMC5838219

[10] Y.-B. Kim , A. Wacker , K. von Laer , V. V. Rogov , B. Suess , H. Schwalbe , Nucleic Acids Res. 2017, 45, 5375. 28115631 10.1093/nar/gkx016 PMC5435998

[11a] R. Hänsel , S. Foldynová-Trantírková , V. Dötsch , L. Trantírek , Top. Curr. Chem. 2013, 330, 47; 22760824 10.1007/128_2012_332

[11b] R. Hänsel , S. Foldynová-Trantírková , F. Löhr , J. Buck , E. Bongartz , E. Bamberg , H. Schwalbe , V. Dötsch , L. Trantírek , J. Am. Chem. Soc. 2009, 131, 15761; 19824671 10.1021/ja9052027

[11c] G. F. Salgado , C. Cazenave , A. Kerkour , J.-L. Mergny , Chem. Sci. 2015, 6, 3314; 28706695 10.1039/c4sc03853c PMC5490339

[11d] M. Krafcikova , R. Hänsel-Hertsch , L. Trantirek , S. Foldynova-Trantirkova , Methods Mol. Biol. 2019, 2035, 397. 31444765 10.1007/978-1-4939-9666-7_25

[12] Y. Yamaoki , A. Kiyoishi , M. Miyake , F. Kano , M. Murata , T. Nagata , M. Katahira , Phys. Chem. Chem. Phys. 2018, 20, 2982. 29022027 10.1039/c7cp05188c

[13] S. Dzatko , M. Krafcikova , R. Hänsel-Hertsch , T. Fessl , R. Fiala , T. Loja , D. Krafcik , J.-L. Mergny , S. Foldynova-Trantirkova , L. Trantirek , Angew. Chem. Int. Ed. 2018, 57, 2165; 10.1002/anie.201712284 PMC5820743 29266664

[14] M. Krafcikova , S. Dzatko , C. Caron , A. Granzhan , R. Fiala , T. Loja , M.-P. Teulade-Fichou , T. Fessl , R. Hänsel-Hertsch , J.-L. Mergny et al., J. Am. Chem. Soc. 2019, 141, 13281. 31394899 10.1021/jacs.9b03031

[15] S. Narasimhan , G. E. Folkers , M. Baldus , ChemPlusChem 2020, 85, 760. 32297474 10.1002/cplu.202000167

[16] E. Luchinat , L. Banci , IUCrJ 2017, 4, 108. 10.1107/S2052252516020625 PMC5330521 28250949

[17a] S. Dzatko , R. Fiala , R. Hänsel-Hertsch , S. Foldynova-Trantirkova , L. Trantirek in New Developments in NMR (Eds.: Ito Y., Dötsch V., Shirakawa M.), Royal Society of Chemistry, Cambridge, 2019, pp. 272–297;

[17b] Y. Yamaoki , T. Nagata , T. Sakamoto , M. Katahira , Biophys. Rev. 2020, 12, 411. 32144741 10.1007/s12551-020-00664-x PMC7242591

[18a] J. N. Kim , A. Roth , R. R. Breaker , Proc. Natl. Acad. Sci. USA 2007, 104, 16092; 17911257 10.1073/pnas.0705884104 PMC1999398

[18b] O. Pikovskaya , A. Polonskaia , D. J. Patel , A. Serganov , Nat. Chem. Biol. 2011, 7, 748. 21841796 10.1038/nchembio.631 PMC3781940

[19] P. Selenko , G. Wagner , J. Struct. Biol. 2007, 158, 244. 17502240 10.1016/j.jsb.2007.04.001

[20] P. Schanda , E. Kupce , B. Brutscher , J. Biomol. NMR 2005, 33, 199. 16341750 10.1007/s10858-005-4425-x

[21] E. Kupce , R. Freeman , J. Magn. Reson. Ser. A 1994, 108, 268.

[22] H. Geen , R. Freeman , J. Magn. Reson. 1991, 93, 93.

[23] S. Ogino , S. Kubo , R. Umemoto , S. Huang , N. Nishida , I. Shimada , J. Am. Chem. Soc. 2009, 131, 10834. 19603816 10.1021/ja904407w

[24] F.-X. Theillet , A. Binolfi , B. Bekei , A. Martorana , H. M. Rose , M. Stuiver , S. Verzini , D. Lorenz , M. van Rossum , D. Goldfarb et al., Nature 2016, 530, 45. 26808899 10.1038/nature16531

[25a] C. Kreutz , H. Kählig , R. Konrat , R. Micura , Angew. Chem. Int. Ed. 2006, 45, 3450; 10.1002/anie.200504174 16622887

[25b] K. Fauster , C. Kreutz , R. Micura , Angew. Chem. Int. Ed. 2012, 51, 13080; 10.1002/anie.201207128 PMC3555429 23161779

[25c] B. Puffer , C. Kreutz , U. Rieder , M.-O. Ebert , R. Konrat , R. Micura , Nucleic Acids Res. 2009, 37, 7728; 19843610 10.1093/nar/gkp862 PMC2794194

[25d] Q. Li , J. Chen , M. Trajkovski , Y. Zhou , C. Fan , K. Lu , P. Tang , X. Su , J. Plavec , Z. Xi et al., J. Am. Chem. Soc. 2020, 142, 4739; 32067454 10.1021/jacs.9b13207

[25e] F. Sochor , R. Silvers , D. Müller , C. Richter , B. Fürtig , H. Schwalbe , J. Biomol. NMR 2016, 64, 63. 26704707 10.1007/s10858-015-0006-9

[26] H.-L. Bao , T. Ishizuka , T. Sakamoto , K. Fujimoto , T. Uechi , N. Kenmochi , Y. Xu , Nucleic Acids Res. 2017, 45, 5501. 28180296 10.1093/nar/gkx109 PMC5435947

