# Supplementary material for: In‐Cell NMR Spectroscopy of Functional Riboswitch Aptamers in Eukaryotic Cells
Source: Angew Chem Int Ed Engl. 2020 Nov 9;60(2):865–72. doi: 10.1002/anie.202007184 (PMC7839747; doi:10.1002/anie.202007184)
Supplement: Supplementary file 1 — Supplementary [file ANIE-60-865-s001.pdf]

Angewandte Chemie
Eine Zeitschrift der Gesellschaft Deutscher Chemiker

Supporting Information

In-Cell NMR Spectroscopy of Functional Riboswitch Aptamers in Eukaryotic Cells

P. Broft, S. Dzatko, M. Krafcikova, A. Wacker, Robert Hänsel-Hertsch, Volker Dötsch, L. Trantirek, and Harald Schwalbe**

anie_202007184_sm_miscellaneous_information.pdf

Supporting Information

Table of Contents

S.1 Material and Methods **2**

RNA preparation of 2'-dG aptamer 70mer and sv-2'-dG aptamer 2

Oocyte and oocyte extract preparation 2

NMR spectroscopy of RNA 2'-dG aptamer 70mer and sv-2'-dG aptamer 3

RNA 14mer transcription template 3

RNA 2'-dG aptamer 72mer transcription template 3

RNA preparation of RNA 14mer and 2'-dG aptamer 72mer 3

FAM-labeled RNA preparation 3

HeLa cells cultivation and preparation 4

In-cell NMR sample preparation 4

Flow cytometry 4

Confocal microscopy 4

(In-cell) NMR spectroscopy of RNA 14mer and 2'-dG aptamer 72mer 5

S.2 Results **5**

Gel experiments 5

In vitro NMR spectra of G-^{15}N-labelled sv-2'-dG aptamer in potassium phosphate buffer 5

Electroporation of HEK and RPE cells with RNA 14mer 6

Confocal microscope images and FCM analysis of the second in-cell NMR experiment of 2'-dG aptamer 72mer 6

Electroporation of HELA cells with 2'-deoxyguanosine 7

References **7**

S.1 Material and Methods

RNA preparation of 2'-dG aptamer 70mer and sv-2'-dG aptamer

^{15}N-labelled nucleotides and ^{13}C, ^{15}N-labeled 2'-deoxyguanosine were purchased from Silantes (Munich). Non-labelled nucleotides were purchased from Sigma (Munich). The 2'-dG aptamer 70mer and the sv-2'-dG aptamer were prepared by *in-vitro* transcription from linearized plasmids using T7-polymerase as described (Stoldt et al.[1], Wacker et al.[2]). RNA concentrations were determined by UV spectroscopy with an extinction coefficient of 6.35*105 M^{-1}*cm^{-1} at 260 nm for the RNA aptamer 70mer and 6.63*105M^{-1}*cm^{-1} for the sequence-modified RNA aptamer. Each RNA oligonucleotide was refolded into a homogeneous conformation by thermal denaturation of the RNA at high concentration (0.2 – 0.5 mM) followed by dilution to 0.05 mM and rapid cooling on ice. RNA samples were exchanged into NMR-buffer (25 mM potassium phosphate, 50 mM potassium chloride at pH 6.2) or intraoocyte buffer (25 mM HEPES, 10.5 mM NaCl, 110 mM KCl, 130 nM $CaCl_2$, 1 mM $MgCl_2$ at pH 7.5) by freeze-drying and subsequent uptake in an appropriate volume of buffer. Conformational homogeneity of all samples was confirmed by native polyacrylamide gel electrophoresis. The RNA-ligand complex for

the RNA aptamer 70mer was prepared at 2-fold molar excess of ligand over RNA. The RNA-ligand complex for the RNA sv-2'-dG aptamer was prepared by further addition of 8-fold molar excess of $MgCl_2$ over RNA. 2'-deoxyguanosine concentrations were determined by UV spectroscopy using a molar extinction coefficient of 1.33 *104 M^{-1}*cm^{-1} at 254 nm.

Oocyte and oocyte extract preparation

X. laevis oocytes and oocyte extract were obtained and prepared as described (Hänsel et al.[3], Krafcikova et al.[4]). *X. laevis* egg maturation was suppressed by addition of progesterone. RNA aptamer and RNA-ligand complex freeze-dried powders were dissolved into 1 mL intraoocyte buffer and concentrated to 2-5 mM stock solutions via a centrifugal ultra-filtration device with 3 kDa cutoff. Care was taken to avoid RNA precipitation. To monitor binding activities and structures of high concentrated RNAs, 7.5 µL of each generated RNA stock solution were diluted twenty-fold with intraoocyte buffer.

NMR spectroscopy of RNA 2'-dG aptamer 70mer and sv-2'-dG aptamer

NMR spectra were recorded at 600 MHz, 700 MHz and 800 MHz Bruker spectrometers equipped with 5 mm $^{1}H(^{13}C/^{15}N)$ cryogenic probes and z-axis gradient systems. 2D [$^{1}H,^{15}N$]-SOFAST-HMQC (Schanda et al.[5]) spectra were recorded at 291 K.

RNA 14mer transcription template

RNA 14mer template (5'-GGC ACC GAA GTG CCT ATA GTG AGT CGT ATT A-3', the underlined region corresponds to the inverse complementary T7 promoter sequence) and T7 promotor (5'-TAA TAC GAC TCA CTA TAG G-3') were purchased from Eurofins Genomics and annealed in a thermocycler at a concentration of 100 µM of each DNA in a reaction volume of 100 µL by incubation at 95 °C for 2 min, followed by cooling the mixture to 59 °C for 1 min and afterwards to 25 °C for 5 min.

RNA 2'-dG aptamer 72mer transcription template

Transcription template for 2'-dG aptamer 72mer (5'-TAA TAC GAC TCA CTA TAG GAA ACT TAT ACA GGG TAG CAT AAT GGG CTA CTG ACC CCG CCT TCA AAC CTA TTT GGA GAC TAT AAG TGA AA-3') was prepared by polymerase chain reaction from a plasmid containing the full-length sequence of the 2'-dG-sensing riboswitch. For increasing of the transcription efficiency an additional G was used at the 5'-end. As forward primer 5'-TAA TAC GAC TCA CTA TAG GAA ACT TAT ACA GGG TAG CA-3' and as reverse primer 5'-[UU]T CAC TTA TAG TCT CCA AAT AG-3' (the nucleotides in brackets are 2'OMe modified) were used. Primers were purchased from Eurofins Genomics. PCR was performed in reaction volumes of 100 µL with 5 µM of each primer, 200 µM of each dNTP, 0.4 ng/mL DNA template and 40 ng Phusion® High-Fidelity DNA Polymerase according to standard protocols from NEB.

RNA preparation of RNA 14mer and 2'-dG aptamer 72mer

RNAs were synthesized by *in vitro* transcription with T7 RNA polymerase from PCR product or DNA template. All transcriptions were optimized for Mg^{2+}, NTPs and DMSO concentrations to achieve maximum yields and homogeneity of the transcripts. For the preparation of unlabelled RNA 14mer a 200 mL transcription was performed and for the preparation of unlabeled 2'-dG aptamer 72mer a 160 mL transcription was performed. For the preparation of FAM-labelled 2'-dG aptamer 72mer a 50 mL transcription was performed. Transcriptions were incubated for 16 h at 37 °C in transcription buffer (200 mM TrisHCl, pH 8.1) with 2 mM spermidine, 5 mM of each NTP, 20-30 mM $Mg(OAc)_2$, 20 mM dithiothreitol (DTT), 0-20 % (v/v) of DMSO , 6 % (v/v) of the PCR mixture or 0.4 µM template and 192 µg T7 RNA polymerase (homemade) in reaction volumes of 10 mL. For the preparation of FAM-labelled 2'-dG aptamer 72mer 30 mM GMP (guanosine 5'-monophosphate disodium salt hydrate) was additional added to the transcription. NTPs were purchased from Carl Roth GmbH (Karlsruhe, Germany) and GMP was purchased from Merck (Darmstadt, Germany). For the removal of precipitated magnesium pyrophosphate during transcription the RNAs were sterile filtered (sterile PES syringe filter 0.45 µm, fisher scientific, Schwerte, Germany). The RNAs were desalted with 200-800 mL ddH_2O using centrifugal concentrators with a molecular weight cut-off of 3000 (Vivaspin 20 from Sartorius AG, Goettingen, Germany) or of 1000 (Microsep Advance from Pall, Port Washington, New York, USA) and purified by denaturing urea PAGE (10-20 % 29:1 (w/w) acrylamide/bisacrylamide, 7 M urea). The RNAs were visualized by UV shadowing (254 nm), excised from the gel, and eluted with ddH_2O at 4°C for 10-34 h. Precipitation was performed with 0.6 M sodium acetate (pH 5.5) and three volumes of ethanol (-20 °C, 16 h).For the removal of acrylamide or further purification the unlabelled RNAs were HPLC purified. 2'-dG aptamer 72mer for FAM-labelling was directly used for the labeling reaction without HPLC-purification. RNA14mer was subjected to standard n-butanol precipitation for desalting and folded by 4 min denaturation at 95 °C at a concentration of 3.75 mM in ddH_20 and cooling to room temperature. 2'-dG aptamer 72mer was desaltet with ddH_20 using centrifugal concentrators as ascribed above and folded by 7 min denaturation at 85 °C at a concentration of 400 µM and immediate diluton to 40 µM with ice cold ddH_20 followed by incubation on ice for 1 h. 2'-dG aptamer 72mer was mixed with 5 eq. of ligand ($^{13}C^{15}N$-labelled 2'-

deoxyguanosine) to obtain the aptamer-ligand complex and lyophilized. The ligand was purchased from Silantes (Munich, Germany). RNA concentration was determined by UV spectroscopy with an extinction coefficient of 733,8 $mM^{-1}*cm^{-1}$ at 260 nm prior to complex formation.

FAM-labelled RNA preparation

5'-FAM-labelled RNA 14mer was purchased from Dharmacon (Lafayette, USA) and 5'-FAM-labelled 2'-dG aptamer 72mer was prepared with monophosphorylated RNA at the 5'-end via EDC-coupling and dye-labeling. EDC-coupling was performed in reaction volumes of 6.5 mL. 1.5 mL of 300 µM (450 nmol, 1 eq.) RNA in EDC-buffer (10 mM K_2HPO_4/KH_2PO_4, pH 7.0; 150 mM NaCl, 10 mM EDTA) were incubated with 250 mg (1.3 mmol, 2889 eq.) EDC ((1-ethyl-3-(3-dimethylaminopropyl)carbodiimide hydrochloride, ThermoFisher Scientific, Waltham, USA); 1 mL freshly prepared imidazol-EDA solution (0.1 M (0.1 mmol 222 eq.) imidazol (pH 6); 0.25 M (0.25 mmol, 556 eq.) EDA) and 4 mL 0.1 M (0.4 mmol 556 eq.) imidazol (pH 6) at 37 °C for 4 h. Afterwards the RNA was precipitated three times with 0.5 M sodium acetate (pH 5.5) and 3 volumes of ethanol. Dye-labeling was performed by incubation of 250 µM RNA and 25 eq of dye (5(6)-SFX (6-(Fluorescein-5-(and-6)-Carboxamido) Hexanoic Acid, Succinimidyl Ester), mixed isomers, ThermoFisher Scientific, Waltham, USA) with 0.05 M $NaHCO_3$ and 50 % DMSO for 40 min at room temperature in reaction volumes of 2 mL. The RNA was precipitated with 0.5 M sodium acetate and three volumes of ethanol, HPLC-purified, and lyophilized.

HeLa cells cultivation and preparation

HeLa cells (Sigma-Aldrich) were cultured in DMEM (without phenol red) (Gibco) supplemented with 10% fetal bovine serum (HyClone, GE Life Sciences) and penicillin-streptomycin solution (100 units penicillin and 0.10 mg streptomycin/mL) (Sigma-Aldrich) under a 5% CO2 atmosphere at 37°C. Prior to electroporation, cells in the late log phase were washed with pre-warmed 1×PBS (Sigma-Aldrich) and harvested using 0.05% trypsin and 0.02% EDTA (Sigma-Aldrich) in 1×PBS. The harvested cells were centrifuged at 1000 rpm for 5 min. Pelleted cells were resuspended in pre-warmed 1×PBS and counted in a Burker counting chamber.

In-cell NMR sample preparation

The preparation of in-cell NMR samples followed protocols described by Viskova et al.[6] and Krafcikova et al.[7] with few modifications. In brief: RNA was integrated into mammalian cells by electroporation using the BTX-ECM 830 system (Harvard Apparatus, USA). RNA 14mer samples for electroporation were prepared by mixing the unlabelled RNA 14mer with FAM-labelled RNA 14mer in a 50:1 ratio in the solution of the electroporation buffer (EB) (1xEB: 140 mM sodium phosphate, 5 mM KCl, 10 mM $MgCl_2$, pH 7.2). Samples of aptamer-ligand-complex for electroporation were prepared by mixing the pre-formed aptamer-ligand complex with FAM-labelled wt-2'-dG aptamer 72mer in a 40:1 ratio in the solution of the electroporation buffer (EB). The final electroporation mixture contained for RNA 14mer 1xEB, 500 µM RNA 14mer and 15 µM FAM-labelled RNA 14mer and for the aptamer-ligand complex 1xEB, 400 µM wt-2'-dG aptamer 72mer, 2 mM ^{13}C, ^{15}N labelled 2'-deoxyguanosine and 10 µM FAM-labelled wt-2'-dG aptamer 72mer. Cells for individual transfections were centrifuged (1000 rpm for 5 min). Pelleted cells were resuspended in the electroporation mixture. Each suspension was divided into 4 mm electroporation cuvettes (Cell Projects Ltd, UK). In each cuvette, 1.6×107 cells were electroporated. All samples were incubated on ice for 5 min prior to electroporation. To achieve maximum transfection efficiency, the electroporation was conducted using two square wave pulses (1000 V/100 µs and 350 V/30 ms) separated by a 5 s interval. After electroporation, the cells were incubated for 2 min at room temperature, transferred into phenol-red-free Leibovitz L15 -/- (no FBS/no antibiotics) medium and centrifuged at 1000 rpm for 5 min to remove the remaining non-incorporated RNA from the cell surface. Cells were resuspended again in the fresh L15 -/- medium. A small fraction of the cell suspension (~6×105 cells) was used for FCM and confocal microscopy analysis to monitor the cell viability and the level of integration and localization of exogenous RNA, respectively. The rest of the cell suspension was centrifuged at 1000 rpm for 5 min. Upon removal of the supernatant, the resulting cell slurry was resuspended in 0.6 mL of Leibovitz L15 -/- containing 10 % of D_2O and transferred into a SHIGEMI NMR tube (Norel, USA). Prior to the NMR measurements, the cells in the NMR tube were manually centrifuged, using a "hand centrifuge" (CortecNet, France), to form a fluffy pellet at the bottom of the NMR tube.

Flow cytometry

For Flow cytometry (FCM) analysis, ~105 cells were resuspended in 200 µL of PBS supplemented with 2 µL of 1 mg/mL of PI (Exbio, Prague, Czech Republic) to stain apoptotic and dead cells or cells with compromised membrane integrity (see below). Then, 104 HeLa cells were analyzed with a BD FACSVerse flow cytometer using the BD FACSuite software (BD Biosciences, San Jose, CA, USA). For the detection of fluorescently (FAM) labelled DNA and the evaluation of transfection efficiency, the excitation wavelength was 488 nm, and the emission was detected at 527/32 nm. PI was excited at 488 nm, and emission was detected at 700/54 nm.

Confocal microscopy

For confocal microscopy, ~5×105 cells were transferred to a 35-mm glass-bottom dish precoated with 0.01% poly-l-lysine (Sigma-Aldrich) supplemented with 2 mL of Leibovitz L15 - /- medium containing 5 µg/mL Hoechst (Sigma-Aldrich) to visualize the nuclei (see below). All microscopy images were obtained using a Zeiss LSM 800 confocal microscope with a 63×/1.2 C-Apochromat objective. Images were taken in transmission mode with 488 nm excitation and emission detection at 480-700 nm for the detection of fluorescently (FAM) labelled DNA. For Hoechst, 405 nm excitation was used with emission detection at 400 to 480 nm.

(In-cell) NMR spectroscopy of RNA 14mer and wt-2'-dG aptamer 72mer

(In-cell) NMR spectra of RNA 14mer were measured at 600 MHz using a Bruker Avance III HD spectrometer equipped with a quadruple-resonance inverse cryogenic probe. The 14mer RNA concentration was 100 µM. The NMR spectra were measured using a JR-echo (1-1 echo) pulse sequence[8] with the zero excitation set to the water resonance and the excitation maximum set to 11 ppm. *In vitro* and in-cell NMR spectra were acquired with 256 with 1280 scans, respectively.
(In-cell) NMR spectra of preformed complex of 2'-dG aptamer 72mer with 2'-dG were measured at 950 MHz using a Bruker Avance III HD spectrometer equipped with a quadruple-resonance cryogenic probe. The RNA concentration of the *in vitro* samples in the 1xEB buffer was 50 µM, unless indicated otherwise. *In vitro* 1D ^{1}H NMR spectra were acquired with 1024 scans using p3919gp pulse sequence (standard Bruker library). In-cell 1D ^{1}H NMR spectra were acquired with 1024 using a JR-echo (1-1 echo) pulse sequence[8] with the zero excitation set to the water resonance and the excitation maximum set to 11 ppm. 1D ^{13}C-edited (in-cell) NMR spectra were acquired using an x-filter 1D based on a SFHMQC[5] with 4096 scans. All the measurements were conducted at 20 ºC. After in-cell NMR spectra acquisition, 1D ^{1}H (^{13}C-edited) NMR spectra of the supernatant from the NMR tube were measured (using the same NMR setup as for in-cell NMR spectrum acquisition) to assess the leakage of transfected aptamer-ligand complex from cells. At the same time, the cells from the NMR tube were subjected to FCM analysis to assess the cell mortality during the course of the NMR experiment.
NMR spectra were processed and analyzed using MNova v12 software (Mestrelab Research, Spain).The *in vitro* and in-cell NMR spectra were processed with the exponential apodization function and the line-broadening of 8 and 14 Hz, respectively.

S.2 Results

Gel experiments

Analytical gel experiments were performed to analyze RNA stability before and after (in-cell) NMR measurements of 2'-dG aptamer 70mer (**Figs. S1A and S1B**) sv-2'-dG aptamer (**Fig. S1B**).

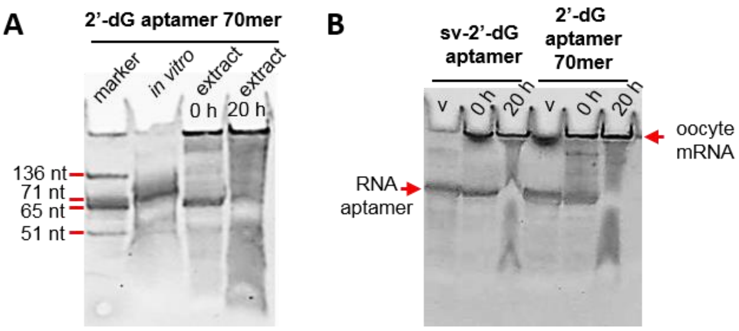

Figure S1. A) Analytical denaturing polyacrylamide gel (12%) of 2'-dG aptamer 70mer before (0h), after (20h) NMR experiment in oocyte cell extract and in oocyte buffer (*in vitro*). **B)** Analytical denaturing polyacrylamide gel (12%) of sv-2'-dG aptamer and 2'-dG aptamer 70mer before (0h), after (20h) in-cell NMR experiment and in oocyte buffer (v).

***In vitro* NMR spectra of G-^{15}N-labelled sv-2'-dG aptamer in potassium phosphate buffer**

2D NMR spectra of G-^{15}N-labelled sv-2'-dG aptamer in the presence and absence of ligand were recorded in potassium phosphate buffer (**Fig. S2**). The resonances of the imino protons of G25 and G32, which are reporter signals for ligand binding, were only visible in the ligand-containing sample.

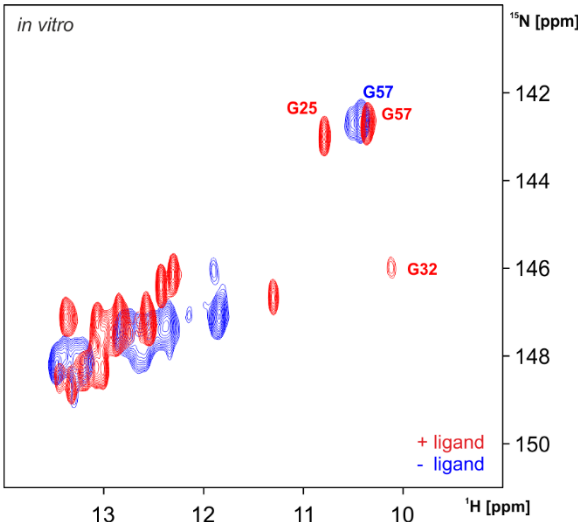

Figure S2. Overlay of the ^{15}N-HMQC spectra of G-^{15}N-labelled sv-2'-dG aptamer in potassium phosphate buffer (25 mM KPi, 50 mM KCl, pH 6.2) in the presence (red) and absence of ligand (blue) at 283 K. The spectrum in the presence of ligand (red) was recorded at 800 MHz and spectrum in the absence of ligand (blue) was recorded at 900 MHz. The resonances of the imino protons of G25 and G32, which are reporter signals for ligand binding, are annotated in red. The resonance of the imino proton of G57, which can be observed in the ligand-bound and ligand-unbound form of the RNA, is annotated in red/blue for the respective sample.

Electroporation of HEK and RPE cells with RNA 14mer

RNA 14mer was delivered into RPE and HEK cells via electroporation. FCM analysis showed that after electroporation more than 80% of the RPE cells and 60% of the HEK cells were viable RNA containing cells and less than 25% of the cells were either dead or had compromised cell membrane integrity (**Fig. S3**).

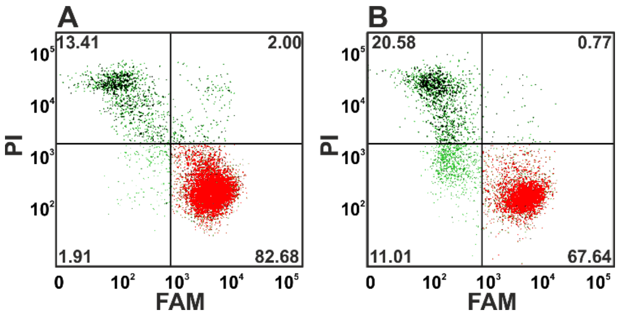

Figure S3. A+B) Double-staining (PI/FAM) FCM analysis of transfected RPE cells (A) and HEK cells (B) with RNA 14mer (FAM). Percentages of viable RNA non-transfected cells, viable RNA-containing cells, non-transfected dead/compromised cells, and transfected dead/compromised cells with RNA are indicated in left-bottom, right-bottom, left-top, and right-top quadrants, respectively.

Confocal microscope images and FCM analysis of the second in-cell NMR experiment of 2'-dG aptamer 72mer

The aptamer-ligand complex was introduced into HeLa cells for a second in-cell NMR experiment. Confocal microscopy indicated that the RNA was homogeneously dispersed all over the cell (**Fig. S4A**). FCM analysis showed that after electroporation more than 90% of the cells were viable RNA containing cells and less than 7% of the cells were either dead or had compromised cell membrane integrity (**Fig. S4B**).

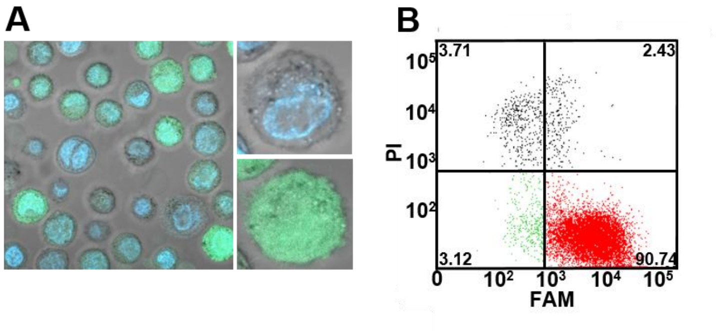

Figure S4. **A)** Confocal microscope images of cells transfected with aptamer-ligand complex. The green color indicates the localization of (FAM)-aptamer/(FAM)-aptamer-ligand complex. The blue color corresponds to a cell nucleus stained by Hoechst 33342. **B)** Double-staining (PI/FAM) FCM analysis of transfected HeLa cells with the aptamer-ligand complex. Percentages of a viable non-transfected cells, viable aptamer-ligand complex containing cells, non-transfected dead/compromised cells, and transfected dead/compromised cells with aptamer-ligand complex are indicated in left-bottom, right-bottom, left-top, and right-top quadrants, respectively.

Electroporation of HELA cells with 2'-deoxyguanosine

HELA cells were electroporated with 400 μM and 2 mM 2'-deoxyguanosine. FCM analysis showed for both concentrations that after electroporation more than 90% of the cells were viable cells (**Fig. S5A and S5B**). Confocal microscopy images taken after electroporation showed intact cells (**Fig. S5C**).

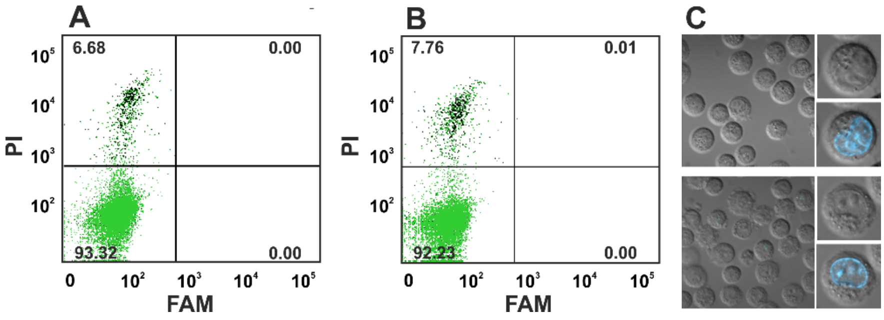

Figure S5. Double-staining (PI/FAM) FCM analysis of transfected HeLa cells with 400 μM **(A)** and 2 mM **(B)** ^{13}C, ^{15}N labelled 2'-deoxyguanosine (ligand). Percentages of viable cells, and dead/compromised cells are indicated in left-bottom, and left-top, respectively. **C)** Confocal microscope images of cells transfected with 400 μM/2 mM (top/bottom) of the ^{13}C, ^{15}N labeled 2'-deoxyguanosine (ligand). The blue color corresponds to a cell nucleus stained by Hoechst 33342.

References

[1] M. Stoldt, J. Wöhnert, M. Görlach, L. R. Brown, *The EMBO journal* **1998**, *17*, 6377.

[2] A. Wacker, J. Buck, D. Mathieu, C. Richter, J. Wöhnert, H. Schwalbe, *Nucleic acids research* **2011**, *39*, 6802.

[3] a) R. Hänsel, S. Foldynová-Trantírková, V. Dötsch, L. Trantírek, *Topics in current chemistry* **2013**, *330*, 47; b) R. Hänsel, S. Foldynová-Trantírková, F. Löhr, J. Buck, E. Bongartz, E. Bamberg, H. Schwalbe, V. Dötsch, L. Trantírek, *Journal of the American Chemical Society* **2009**, *131*, 15761.

[4] M. Krafcikova, R. Hänsel-Hertsch, L. Trantirek, S. Foldynova-Trantirkova, *Methods in molecular biology (Clifton, N.J.)* **2019**, *2035*, 397.

[5] P. Schanda, E. Kupce, B. Brutscher, *Journal of biomolecular NMR* **2005**, *33*, 199.

[6] P. Viskova, D. Krafcik, L. Trantirek, S. Foldynova-Trantirkova, *Current protocols in nucleic acid chemistry* **2019**, *76*, e71.

[7] M. Krafcikova, S. Dzatko, C. Caron, A. Granzhan, R. Fiala, T. Loja, M.-P. Teulade-Fichou, T. Fessl, R. Hänsel-Hertsch, J.-L. Mergny et al., *Journal of the American Chemical Society* **2019**, *141*, 13281.

[8] V. Sklenář, A. Bax, *Journal of Magnetic Resonance (1969)* **1987**, *74*, 469.

Author Contributions

P.B., A.W., S. D., M. K. R.H.H. have conducted the experiments, P.B., A.W., R.H.H., L.T., H.S. have designed the experiments, P.B., A.W., L.T., H.S. have written the original draft. L.T., H.S. have obtained funding for conducting this research.
